# Mapping quantitative trait loci for yield-related traits and predicting candidate genes for grain weight in maize

**DOI:** 10.1038/s41598-019-52222-5

**Published:** 2019-11-06

**Authors:** Yanming Zhao, Chengfu Su

**Affiliations:** College of Agronomy, Qingdao Agricultural University, Qingdao, 266109 P.R. China

**Keywords:** Genetic linkage study, Plant breeding

## Abstract

Quantitative trait loci (QTLs) mapped in different genetic populations are of great significance for marker-assisted breeding. In this study, an F_2:3_ population were developed from the crossing of two maize inbred lines SG-5 and SG-7 and applied to QTL mapping for seven yield-related traits. The seven traits included 100-kernel weight, ear length, ear diameter, cob diameter, kernel row number, ear weight, and grain weight per plant. Based on an ultra-high density linkage map, a total of thirty-three QTLs were detected for the seven studied traits with composite interval mapping (CIM) method, and fifty-four QTLs were indentified with genome-wide composite interval mapping (GCIM) methods. For these QTLs, Fourteen were both detected by CIM and GCIM methods. Besides, eight of the thirty QTLs detected by CIM were identical to those previously mapped using a F_2_ population (generating from the same cross as the mapping population in this study), and fifteen were identical to the reported QTLs in other recent studies. For the fifty-four QTLs detected by GCIM, five of them were consistent with the QTLs mapped in the F_2_ population of SG-5 × SG-7, and twenty one had been reported in other recent studies. The stable QTLs associated with grain weight were located on maize chromosomes 2, 5, 7, and 9. In addition, differentially expressed genes (DEGs) between SG-5 and SG-7 were obtained from the transcriptomic profiling of grain at different developmental stages and overlaid onto the stable QTLs intervals to predict candidate genes for grain weight in maize. In the physical intervals of confirmed QTLs *qKW-7*, *qEW-9, qEW-10*, *qGWP-6*, *qGWP-8*, *qGWP-10*, *qGWP-11 and qGWP-12*, there were 213 DEGs in total. Finally, eight genes were predicted as candidate genes for grain size/weight. In summary, the stable QTLs would be reliable and the candidate genes predicted would be benefit for maker assisted breeding or cloning.

## Introduction

Maize is a very important crop which plays an important role in food, animal feed and the raw materials of bioenergy worldwide^1^. Obtaining high grain yield is of great significance for maize breeders. Yield-related traits are all complex quantitative traits, controlled by multiple genes. It is difficult to explain yield formation mechanism just from maize phenotypies. To improve maize yield, it is important to study on the relations between yield and yield-related traits at molecular level. Since Helentjaris and Slocum *et al*. published the first piece of molecular marker linkage map of maize in 1986^2^, large amounts of QTLs have been mapped for yield traits^3–6^. To date, QTL mapping methods have been successfully used in maize and many QTLs associated with maize yield-related traits were identified. The identified QTLs included 36, 45, 149, 46 and 23 associated with cob diameter, ear diameter, grain weight, ear length and kernel row number, respectively (https://archive.gramene.org/qtl/).

In earlier plant breeding, researchers directly utilized the markers linked with identified QTLs in marker assisted breeding to enhance breeding efficiency^7^. However, QTL mapping results are usually vary among different experimental materials and different experimental environments^8^. Thus, it is important for QTLs to be confirmed or to be fine mapped before used for marker assisted breeding^9^. For QTL confirmation, one scheme is to detect QTLs in early generations like F_2_ and F_2:3_, and then confirmed in advanced generations from the same cross. Such approach have been successfully conducted in rice^9^, sweet sorghum^10^, soybean^11^, cucumber^12^. And the early generations with beneficial effect would be served as new breeding materials for cultivating new varieties^7^. Another scheme for confirming QTLs is to analyze if the target QTLs are stable and common between or among different populations. For example, the trichome density trait was confirmed with four recombinant inbred lines (RIL) populations of *A. thaliana*^13^*, the* stay green trait was confirmed with two RILs populations of sorghum^14^, and the kernel length trait of barley was confirmed by different RILs population^15^.

Grain weight is one of the most important yield-related trait in crops. It is of great significance to clone the genes controlling grain weight and then to clarify their molecular genetic mechanism. Great achievements have been made in genes/QTLs identification and dissection for grain size and grain weight in many crops, such as rice^16–20^, soybean^21,22^, wheat^23,24^. Especially many genes related to grain weight or grain size in rice, including *GS3*^25^, *GS5*^16^, *qGL3*^26^, *GW2*^18^, *GW8*^27^, *GS2*^28^, *qGW7/GL7*^29^, have been successfully cloned. The evidence showed that the grain size trait was regulated by multiple signaling pathways, and the main pathway included ubiquitin-proteasome degradation pathway^30^, phytohormone signaling pathway and G protein independent pathway. Earlier studies showed that crop’s yield was greatly influenced by grain size and grain weight. Although great achievements on the genes controlling grain size and grain weight have been made in maize in recent years^5,31–33^, it was relatively low compared to rice and *Arabidopsis thaliana*. It is necessary for maize yield-related traits to confirm stable QTLs, to decrease functional gene number in stable QTL intervals and to predict candidate genes. All these works would provide basis for cloning functional genes and marker-assisted breeding.

The purposes of this study were: (1) to identify QTLs for yield-related traits with an F_2:3_ population from SG5 × SG7; (2) to compared these QTLs detected in this study with the QTLs identified in other populations, including an F_2_ population from the same parents as the F_2:3_ used in this study and recent studies; (3) RNA-seq technology was applied to identify transcriptional variations between maize inbred lines SG5 and SG7 subjected to grain weight; (4) to identify the DEGs related to grain weight between SG5 and SG7 and to predict candidate genes.

## Results

### Phenotype evaluation of the mapping population

The phenotypic data of seven yield traits, i.e., ear length (EAL), ear diameter (EAD), cob diameter (CD), kernel row number (KRN), ear weight (EW), grain weight per ear (GWP) and 100-kernel weight (KW), were collected from the F_2:3_ mapping population in 2016. The mean values of the seven traits were shown in Table 1. The phenotypic values of the two parents SG5 and SG7 were different in all the seven traits. The seven yield traits all displayed in bell-shaped normal distribution (Supplemental Fig. 1). Table 2 listed the pearson’s correlation coefficients and the significance tests between every two of the seven observed traits. The closes relation occurs between GWP and EW (0.975).

**Table 1:** Descriptive statistics of traits in the F_2:3_ mapping population of maize derived from the cross of SG5 and SG7.

Trait	SG5(P_1_)	SG7(P_2_)	Min	Max	Mean	Std.Dev.
Ear length (cm)	14.28	12.69	12.82	21.5	15.67	1.34
Ear diameter (cm)	3.71	5.03	3.54	5.47	4.6	0.33
Cob diameter (cm)	2.54	3.27	2.29	3.96	3.05	0.23
Kernel row number	8	16	9	16	12.08	1.26
Grain number per row	23.41	17.19	18	38	28.53	3.39
Ear weight (g)	143.08	73.11	52.813	280.3	128.639	22.47
Grain weight per cob (g)	112.01	21.16	31.788	218.4	97.548	19.85
100-kernel weight (g)	35.005	25.72	20.521	44.03	33.604	3.53

**Table 2:** Pearson correlations for yield related traits of maize from the F_2:3_ population of SG5 × SG7.

Trait	EAL	EAD	CD	KRN	EW	GWP	KW
EAL	1						
EAD	−0.005	1					
CD	−0.021	0.573**	1				
KRN	−0.014	0.206**	0.219**	1			
EW	0.350**	0.601**	0.392**	0.240**	1		
GWP	0.307**	0.558**	0.311**	0.240**	0.975**	1	
KW	0.333**	0.259**	0.149*	−0.171*	0.554**	0.545**	1

*Significantly different from 0 at alpha = 0.05;

**Significantly different from 0 at alpha = 0.01.

### QTL analysis using a high-density linkage map

**Figure 1:**
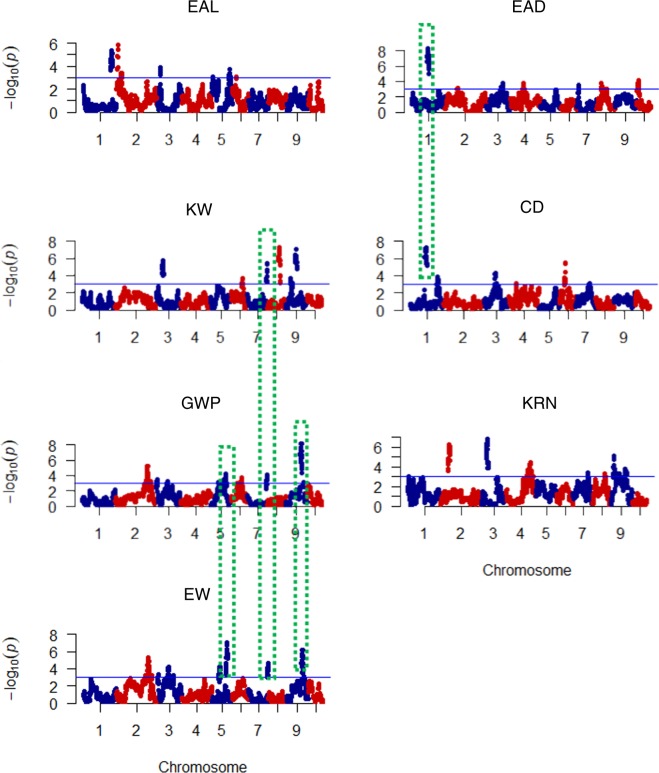
Plots of test statistic −Log10(p) against genome location for seven traits of maize using the CIM method. The horizontal blue line of each panel is the critical value of the test statistic. The seven traits are: 100-kernel weight (KW), ear length (EAL), ear diameter (EAD), cob diameter (CD), kernel row number (KRN), ear weight (EW), and grain yield per plant (GWP). Dotted rectangle with green color indicate pleiotropism phenomenon might exist.

CIM and GCIM procedures were applied to identify QTLs associated with the seven yield traits based on a high-density linkage map. The map constructed in previous study^34^, had 3305 bin-markers. Using CIM, a total of thirty-three QTLs were detected for the seven yield traits. These QTLs were distributed on all of the 10 maize chromosomes. Among them, four QTLs controlled KW trait and were located on maize chromosomes 3, 7, 8, and 9; five related to EAL trait were located on chromosomes 1, 2, 3, and 5; four associated with EAD trait were located on chromosomes 1, 4, and 10; four controlling CD trait were located on chromosomes 1, 3, and 6; five controlled KRN trait and were located on chromosomes 2, 3, 4, and 9; four were associated with EW trait and located on 2, 5, 7, and 9; and seven controlling GWP trait were located on chromosomes 2, 3, 5, 7, and 9. QTL mapping result from CIM procedure is shown in Fig. 1 and Table 3. The physical intervals of 33 QTLs ranged from 0.15 to 23.75 Mb according to reference genome. The phenotypic variation explained by single QTL ranged from 4.5 to 25.6%, the means of KW, EAL, EAD, CD, KRN, EW, and GWP being 7.28, 12.2, 10.15, 12.2, 9.56, 10.4, and 9.6%, respectively. The logarithm of odds (LOD) scores ranged from 3.0 (*qKW-12*) to 7.4 (*qEAD1-1*).

**Table 3:** QTL identified for seven yield  traits of maize using high-density SNP bin-map from composite interval mapping (CIM).

Trait	QTL	Chr	Positions (Mb)	Interval (Mb)	Physical length (Mb)	LOD	ADD^a^	DOM^b^	R^2^(%)	QTL-MI^c^ (Mb)	References
KW	*qKW-11*	3	25.65	25.15–26.2	1.05	4.8	−1.80	0.40	10.5		
	*qKW-7*	7	175.15	174.05–175.6	1.55	4.5	−1.05	−0.62	7.3	170.3–176.2	Chen *et al*., 2017
	*qKW-13*	8	158.85	153.3–159.6	6.3	6.5	−1.47	−0.45	8.4	146.9–160.5	Chen *et al*., 2017
	*qKW-14*	9	68.45	66.9–71.1	4.2	6.2	1.23	0.63	5.7		
EAL	*qEAL-7*	1	275.1	274.6–279.55	4.95	5.1	−3.88	−3.43	9.8		
	*qEAL-8*	2	3.25	3.15–3.3	0.15	5.1	−0.01	−4.69	25.6	3.2–3.3	Xiao *et al*., 2016
	*qEAL-9*	1	279.7	279.55–280.85	1.3	4.3	−3.25	−4.13	9.2	279.3–279.6	Xiao *et al*., 2016
	*qEAL-4*	3	4.95	3.15–5.45	2.3	3.2	−0.86	−4.66	6.4	5.8–6.0	Xiao *et al*., 2016
	*qEAL-10*	5	211.2	208.35–211.75	3.4	3.0	6.58	−3.78	10.0		
EAD	*qEAD-1*	1	197.75	197.45–199.25	1.8	6.6	4.65	1.71	10.4		
	*qEAD1-1*	1	201.25	199.25–202.6	3.35	7.4	5.35	0.82	12.0		
	*qEAD-8*	4	53.6	50.7–56.55	5.85	3.0	4.79	−3.52	9.7		
	*qEAD-9*	10	26.5	14.5–28.95	14.45	3.4	−4.54	4.93	8.5		
CD	*qCD-3*	1	197.2	196.85–197.45	0.6	6.4	3.00	1.10	9.7		
	*qCD3-1*	1	201.25	200.85–205.3	4.45	5.3	3.07	0.64	9.9		
	*qCD-5*	3	115.25	115.05–125.65	10.6	3.6	−3.60	1.78	12.4		
	*qCD-6*	6	88.1	82.3–102.75	20.45	4.7	−4.62	2.10	16.8		
KRN	*qKRN2-1*	2	18.15	13.6–21.1	7.5	5.5	0.43	0.29	5.5	18.5–18.6	Xiao *et al*., 2016
	*qKRN-2*	2	23.45	21.1–24.35	3.25	5.2	0.47	0.16	7.2		
	*qKRN-4*	3	20.05	16.55–25.15	8.6	6.0	0.90	−0.18	15.0		
	*qKRN-5*	4	205.95	205.7–206.35	0.65	3.7	0.51	−0.65	7.1		
	*qKRN-6*	9	13.95	13.35–14.25	0.9	4.4	0.78	−0.40	13.0		
*EW*	*qEW-7*	2	211.7	209.3–212.45	3.15	4.5	−11.28	9.60	12.9		
	*qEW-8*	5	194.0	192.95–195.85	2.9	3.5	0.20	8.46	6.9		
	*qEW-9*	7	174.45	174.05–175.6	1.55	3.4	−4.22	−5.26	6.8	170.3–176.2	Chen *et al*., 2017
	*qEW-10*	9	136.3	135.75–138.6	2.85	7.3	4.54	8.48	15.0	136.8–142.6	Xiao *et al*., 2016
GWP	*qGWP-6*	2	206.95	199.85–209.8	9.95	4.5	−13.35	9.96	13.3	206.2–206.7	Xiao *et al*. 2016
	*qGWP-7*	3	114.90	111.9–117.85	5.95	3.5	−10.55	2.22	9.9		
	*qGWP-8*	5	189.45	188.2–190.75	2.55	3.9	5.27	5.47	7.7	188.8–190.2	Xiao *et al*., 2016
	*qGWP-9*	5	194.0	192.95–195.85	2.9	6.2	5.95	7.71	11.8		
	*qGWP-10*	7	174.6	174.05–175.6	1.55	3.9	−4.07	−6.41	7.3	170.3–176.2	Chen *et al*., 2017
	*qGWP-11*	9	136.3	135.75–138.6	2.85	5.4	2.02	9.75	10.0	136.8–142.6	Xiao *et al*., 2016
	*qGWP-12*	9	142.35	1420.5–143.65	1.6	3.8	0.44	9.21	7.2	136.8–142.6	Xiao *et al*., 2016

^a^Estimated additive effect.

^b^Estimated dominance effect.

^c^Marker interval of QTLs identified in previous studies.

The same below.

For GCIM, a total of fifty-four QTLs were detected. Among these QTLs, fourteen were also detected by CIM (Table 4), three KW QTLs were located on chromosomes 7, 8 and 9, two EAD QTLs on chromosomes 1, and 2, nine CD QTLs on chromosomes 1, 2, 4, 5, 6, and 7, thirty-four KRN QTLs on over all chromosomes except chromosomes 6 and 9, two EW QTLs on chromosomes 2 and 7, and four GWP QTLs on chromosomes 5, 7, and 9. Their LOD scores ranged from 3.1 (*qKW-7*) to 19.4 (*qKRN-8*). The GCIM approach seems more powerful in detecting small effects QTLs, especially for early generation population. The related information is summarized in Table 4

**Table 4:** QTL identified for seven traits of maize using high-density SNP bin-map from the GCIM method.

Trait	QTL	Chr	Position (Mb)	ADD	DOM	LOD	CIM^k^	R^2^(%)	QTL-MI(Mb)	References
KW	*qKW-7*	7	176.10	−0.81	0.00	3.1	Yes	2.67	170.3–176.2	Chen *et al*., 2017
	*qKW-13*	8	158.85	−1.21	0.00	5.2	Yes	5.85	146.9–160.5	Chen *et al*., 2017
	*qKW-14*	9	68.65	1.47	0.00	6.7	Yes	8.67		
EAD	*qEAD1-1*	1	201.25	2.09	0.00	3.4	Yes	2.22		
	*qEAD10*	2	3.30	0.00	2.68	3.9		1.82		
CD	*qCD-7*	1	195.60	0.00	1.81	4.5	Yes	1.71		
	*qCD-8*	1	216.45	1.76	0.00	4.6		3.25		
	*qCD-9*	2	58.25	2.19	0.00	6.1		5.02		
	*qCD-3*	2	194.35	−3.17	0.00	8.9	Yes	10.50		
	*qCD-10*	4	206.00	1.48	0.00	3.5		2.29		
	*qCD-11*	4	230.75	0.00	−2.38	5.6		2.96		
	*qCD-12*	5	205.80	1.80	0.00	4.0		3.39	204.5	Xue *et al*., 2013
	*qCD-13*	6	12.25	−2.05	0.00	4.6		4.41		
	*qCD-14*	7	129.35	1.70	0.00	4.3		3.03		
KRN	*qKRN-7*	1	56.35	0.00	−0.14	3.4		0.33		
	*qKRN-8*	1	196.20	0.00	0.49	19.4		3.78	196–197.4	Xiao *et al*., 2016
	*qKRN-9*	1	249.05	0.00	−0.23	5.3		0.81	250.0–254.4	Chen *et al*., 2017
	*qKRN-10*	1	287.70	0.00	−0.17	3.8		0.45		
	*qKRN-11*	2	6.25	0.00	0.25	7.1		0.97		
	*qKRN2-1*	2	18.85	0.41	0.00	14.4	Yes	5.20	18.5–18.6	Xiao *et al*., 2016
	*qKRN-12*	2	137.40	0.00	0.23	5.8		0.86		
	*qKRN-13*	2	192.45	0.00	−0.33	9.5		1.72	195.5–195.7	Xiao *et al*., 2016
	*qKRN-14*	2	197.20	−0.28	0.00	7.2		2.46	198.2–202.2	Xiao *et al*., 2016
	*qKRN-15*	3	2.65	0.00	0.31	9.9		1.50		
	*qKRN-16*	3	6.25	0.00	−0.46	18.2		3.35		
	*qKRN-17*	3	10.60	0.49	0.00	19.2		7.62	7.67–10.08	Chen *et al*., 2017
	*qKRN-18*	3	180.35	−0.25	0.00	6.1		2.02	178.1–183.9	Chen *et al*., 2017
	*qKRN-19*	3	221.55	0.00	−0.16	3.7		0.43	221.6–222.6	Chen *et al*., 2017
	*qKRN-20*	4	1.15	0.00	−0.17	3.3		0.45		
	*qKRN-21*	4	49.15	0.00	0.26	7.5		1.05		
	*qKRN-22*	4	177.85	0.18	0.00	4.1		0.98		
	*qKRN-23*	4	179.05	0.00	−0.15	3.6		0.37		
	*qKRN-5*	4	207.40	0.00	−0.34	11.5	Yes	1.84		
	*qKRN-24*	4	238.10	0.00	−0.28	7.3		1.21		
	*qKRN-25*	5	16.80	0.21	0.00	5.2		1.35	16.4–16.9	Xiao *et al*., 2016
	*qKRN-26*	5	205.80	0.22	0.00	5.3		1.47	205.4–207.7	Chen *et al*., 2017
	*qKRN-27*	7	31.45	−0.24	0.00	7.9		1.86	19.44–34.19	Chen *et al*., 2017
	*qKRN-28*	7	157.45	0.00	−0.38	14.0		2.29		
	*qKRN-29*	8	161.05	0.15	0.00	4.3		0.74	160.3–163.3	Chen *et al*., 2017
	*qKRN-30*	8	165.55	0.00	−0.37	12.4		2.16	163.9–164.1	Xiao *et al*., 2016
	*qKRN-31*	8	170.55	0.00	0.35	12.1		1.97	170.3–170.7	Xiao *et al*., 2016
	*qKRN-32*	9	12.45	0.00	−0.27	8.0	Yes	1.19		
	*qKRN-6*	9	14.05	0.47	0.00	17.5	Yes	6.98		
	*qKRN-34*	9	26.75	0.00	−0.18	4.5		0.54		
	*qKRN-35*	9	127.65	0.00	−0.18	4.3		0.50		
	*qKRN-36*	9	142.05	0.20	0.00	4.9		1.21		
	*qKRN-37*	9	154.25	0.00	0.27	8.3		1.12		
	*qKRN-38*	9	155.05	0.19	0.00	4.1		1.11		
GWP	*qGWP-13*	5	44.00	−5.48	0.00	3.3		2.98	38.17–61.53	Chen *et al*., 2017
	*qGWP-9*	5	197.10	6.51	0.00	4.3	Yes	4.20		
	*qGWP-10*	7	176.35	−5.73	0.00	3.6	Yes	3.25	170.3–176.2	Chen *et al*., 2017
	*qGWP-14*	9	108.05	5.81	0.00	3.5		3.34	90.8–107.9	Chen *et al*., 2017
EW	*qEW-7*	2	211.70	0.00	5.99	4.1	Yes	2.28		
	*qEW-9*	7	174.45	−5.63	0.00	3.5	Yes	4.01	170.3–176.2	Chen *et al*., 2017

^k^Indicates whether or not the QTL has been identified by the CIM method.

.

### Confirmation of QTLs in different generation materials

**Figure 2:**
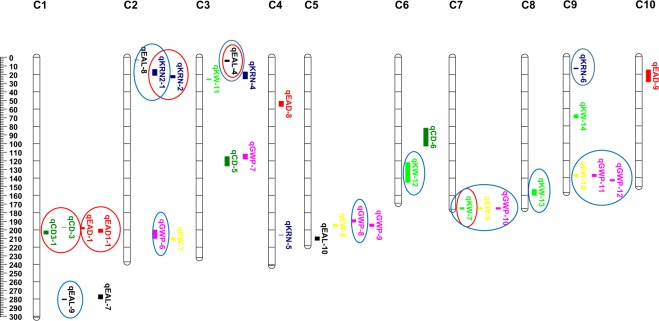
QTL locations for seven yield-related traits studied in the SG5/SG7 F_2:3_ population. QTLs were represented in different colors for seven traits including blue for EAD (ear diameter, mm), pink for CD (cob diameter, mm), green for EAL (ear length, mm), brown for KRN (kernel row number), light blue for EW (ear weight, g), yellow for GWP (grain weight per ear) and red for KW (100-kernel weight, g) on chromosomes C1 to C10. QTLs represented by bars are shown on the right of the linkage groups, close to their corresponding markers. Supported intervals for each QTL are indicated by the length of vertical bars. The eight QTLs circled in red were stably detected in both F_2_ and F_2:3_ populations with CIM. The fifteen QTLs circled in blue were also detected in same or similar physical positions by previous studies with CIM.

It is essential to confirm QTLs before used in marker assisted breeding^35^. We compared the QTLs mapped with the F_2:3_ population with those detected using other populations, including an F_2_ generating the same parents as the mapping population in this study^34^. For the 33 QTLs mapped by CIM in this study (Table 3), eight of them located on chromosomes 1, 2, 3, and 7 were consistent with those detected in the F_2_ population (Fig. 2). There were fifteen major QTLs overlapped with the those identified in previous studies^36–38^ (Fig. 2), and they were located on chromosomes 1, 2, 3, 5, 7 and 9. For the fifty-four QTLs detected by GCIM, five of them(*qKW-7, qEAD1-1, qCD-3, qKRN2-1, qKRN-1*) were also mapped in the F_2_ population from the same parents, and twenty one were identical to the reported QTLs in other studies (Table 4). The QTL detected in more than one mapping populations is considered to be stable QTL.

To sum up, for all the QTLs detected by CIM and GCIM methods, thirty of them were stable QTLs, and fourteen and twenty-two were confirmed by CIM and GCIM, respectively. The detailed information about confirmed QTLs were listed in Tables 3 and 4. The confirmed QTLs for grain weight include one major QTL for KW on chromosome 7 (*qKW-7*, mapped by CIM and GCIM), two major QTLs for EW on chromosome 7 and 9 respectively (*qEW-9*, mapped by CIM and GCIM; and *qEW-10*, mapped by CIM only), two major QTLs for GWP on chromosome 2 and 7 respectively (*qGWP-6*, mapped by CIM only; *qGWP-10*, mapped by CIM and GCIM), two major QTLs for GWP on chromosome 5 (*qGWP-8* and *qGWP-13*, detected by CIM and GCIM respectively), and three major QTLs for GWP on chromosome 9 (*qGWP-11* and *qGWP-12*, mapped by CIM; and *qGWP-14*, mapped by GCIM).

### Comparison of QTL regions

In this study, a total of thirty-three QTLs and fifty-four QTLs were respectively mapped by using CIM and GCIM methods for the seven yield-related traits. However, more than half of these QTLs were not stable, implying that these traits were controlled by multiple minor genes. The closely connected QTLs might be one locus, such as*qEAD1* and *qEAD1-1* on chromosome 1*, qCD-3* and *qCD3-1* on chromosome 1*, qKRN-2* and *qKRN-2-1* on chromosome 2. In addition, some of the detected QTLs in this study tended to have pleiotropism phenomenon. For example, *qEAD-1* and *qCD-3* were located on the same position on chromosome 1, but they were related to EAD and CD respectively. *qEW-8 and qGWP-9* were located on the same position on chromosome 5, but they were related to EW and GWP respectively. *qEW-10* and *qGWP-11* located on the same position on chromosome 9 were related to EW and GWP respectively. And *qKW-7*, *qEW-9* and qGWP-*10 were* located on the same position on chromosome 7, but they were related to KW, EW and GWP respectively.

### Identification of candidate DEG for grain weight

RNA-seq procedure was carried out on an Illumina NovaSeq instrument. Totally, 18 RNA samples from SG5 and SG7 were collected form grains at three developmental stages (three biological replicates per stage) and used for RNA-seq analysis. The Pearson correlation analysis for RNA-seq data between samples was showed in Supplemental Table 2 and Fig. 2. A total of 932,738,890 with average 51, 818,827 clean reads on each chromosome were obtained after we filtering and performing quality control against the raw reads. (Supplemental Table 1). The clean reads were mapped onto the maize B73 genome by TopHat v. 2.0.12^39^ software. For each replicate, 40,277,620–62,763,408 reads were obtained. Of these reads, 81.97–86.23% were mapped to the reference genome as unique and multiple matches (Supplemental Table 1). Based on these mapped reads, DEGs, novel transcripts, alternative splicing and events were detected. The fragments per kilobase of exon per million fragments mapped (FPKM) model was used for calculating all genes expression values. The threshold of corrected P-value 0.05 and log_2_ (Fold change) of 0.5 (absolute value) were set as the thresholds for pair-wise comparison to detect DEGs between SG5 and SG7. To analyze the functions of DEGs, Gene Ontology function enrichment was conducted by Blast swiss prot database (website). The top significant biological process (BP), cellular component (CC), and molecular function (MF) terms were shown in Supplemental Fig. 3. In the BP group, DEGs are enriched in carbohydrate metabolic and lipid metabolic processes. In the MF group, DEGs are enriched in catalytic activity, hydrolase activity, oxidoreductase activity and so on. In the CC group, DEGs enrichment do not reach up to a significant level.

To decrease the candidate DEG number, we focused on those overlapped within the physical intervals of those confirmed QTLs related to grain weight. These QTLs were located on chromosomes 1, 2, 5, 7, and 9, including *qKW-7, qEW-9, qEW-10, qGWP-6, qGWP-8, qGWP-10, qGWP-11, qGWP-12, qGWP-13* and *qGWP-14*. There were more than 1000 protein-coding genes in the mapped physical intervals in total, but after removing those genes that were not DEGs between parents, only 213 genes left and applied for further comparative genomics analysis and candidate gene prediction (Supplemental Table 3).

### Candidate gene prediction

In ubiquitin-proteasome degradation pathway, grain size/weight was regulated by the interactions among three kinds of protein enzymes, i.e., ubiquitin protein ligase E3, ubiquitin-binding enzyme E2 and ubiquitin-activating enzyme E1^30^.The functional mechanism of *DA2*^40^ in *Arabidopsi*s and *GW2*^18^ in rice in regulating grain size were both involved in the ubiquitin-proteasome degradation pathways. Previous studies have proved that abscisic acid (ABA) plays functions during seed development, and ABA-deficient *Arabidopsis* mutants produce seeds with increased size and mass^41^. MADS-box transcription factor genes have also been proven to play critical role in regulating grain size/weight indirectly^42^. FEM111/AGL80 is such a kind of gene in *Arabidopsi*s^43^. Among the candidate genes in the intervals of *qGWP-6*, there were two genes GRMZM2G097089 and GRMZM2G158191 encoding E3 ubiquitin protein ligase. At *qGWP-8* interval, one gene GRMZM2G169994 encoding E3 ubiquitin protein ligase was located. At the physical interval of the three identical QTLs *qKW-7*, *qEW-9* and *qGWP-10*, there were two genes, one was GRMZM2G113039 that encodes E3 ubiquitin protein ligase and the other was GRMZM2G134480 that encodes ubiquitin-activating enzyme E1. At the physical interval of the two consistent QTLs *qEW-10* and *qGWP-11*, two genes GRMZM2G057959 and GRMZM2G128953 were located and encoded ABA receptor MADS-box transcription factor respectively. And at *qGWP-12* interval, one gene GRMZM2G036697 that encodes probable E3 ubiquitin-protein ligase was located.

These eight genes were chosen as candidate genes for grain weight in maize for the future prospects (Table 5).

**Table 5:** Predicted candidate genes for grain weight.

geneID	Chr	Start(bp)^h^	End(bp)^m^	length(bp)^n^	Annotation from Blast swiss prot	Correlated QTLs	logFC^p^ or RCP1/RCP2^q^		
							Day5	Day10	Day15
GRMZM2G097089	2	200141982	200159406	2125	E3 ubiquitin protein ligase RIN2	*qGWP-6*	0.48	0.63	0.34
GRMZM2G158191	2	201401772	201408548	899	E3 ubiquitin-protein ligase NEURL1B	*qGWP-6*	10.45/0	4.68	4.34/0
GRMZM2G169994	5	188882081	188886562	1634	E3 ubiquitin-protein ligase RMA1H1	*qGWP-8*	−1.00	0.03	0.03
GRMZM2G113039	7	174555103	174558004	1609	E3 ubiquitin/ISG15 ligase TRIM25	*qKW-7*/*qEW-9*/*qGWP-10*	2.22	2.05	2.51
GRMZM2G134480	7	174785186	174790941	3469	Ubiquitin-activating enzyme E1 3	*qKW-7*/*qEW-9*/*qGWP-10*	−0.44	−0.50	−1.32
GRMZM2G057959	9	137279301	137280649	1349	Abscisic acid receptor PYL4	*qEW-10*/*qGWP-11*	−1.29	1.15	1.73
GRMZM2G128953	9	138090540	138096148	1130	MADS-box transcription factor 31	*qEW-10*/*qGWP-11*	3.70	2.92	0.09
GRMZM2G036697	9	142186954	142191153	2008	Probable E3 ubiquitin-protein ligase LOG2	*qGWP-12*	−0.67	−0.30	−0.05

^h^Start physical position of the gene;

^m^End physical position of the gene;

^n^Gene lenth;

^p^LogFC: Log 2 ratio, number of folds the gene is differentially expressed in RNA-seq;

^q^RCP1/RCP2: different of readcounts between P_1_ and P_2_;

Positive sign of logFC indicates gene transcript expressed high in SG5 while negative sign indicates gene transcript expressed high in SG7.

## Discussion

High yield is a permanent objective for maize breeders. Studies on QTL mapping and gene dissection for yield-related traits have become a research focus in maize in recent years^37,44–46^. It is of great significance for maize breeder to map QTLs, to predict candidate genes, to clone functionlal genes and to clarify gene’s genetic mechanism for yield-related traits, especially for grain weight. Compared to rice and *Arabidopsis thaliana*, the studies on maize grain weight candidate genes were relatively slow. Stable QTLs are useful for marker-assisted breeding. QTLs mapped in one population might well be not detected in another population. Thus, it is critical important for QTLs to be confirmed to rule out statistical anomalies while used in marker assisted breeding^7^. To validate QTLs, the first scheme is to confirm them in other mapping populations, the second scheme is to confirm them in different generations from the same crossing, and the third is to confirm QTLs using the same population evaluated in multiple locations and in multiple years. Mapping QTLs in early generations is that the QTLs with beneficial effect for breeding lines in early generations can be transferred to the high generations directly. In addition, the QTLs detected in early generation are more than those in late generation, especially for those minor QTLs^47^. In this study, an F_2:3_ population developed from the cross between SG-5 and SG-7 was used for mapping QTLs for seven yield-related traits. In terms of phenotypic segregation issue in F_2:3_, Zhang *et al*. (2004) proposed to combine the F_2_ phenotypes with the F_2:3_ average phenotypes to further increase the power of QTL mapping^48^. In this study, the F_2:3_ phenotypic value were the mean value of eight F_2:3_ individuals of F_3_ progeny derived from F_2_ plant selfing. The confirmed QTLs for yield-related traits are considered as stable QTLs and could be applied in marker assisted breeding, gene cloning and function analyzing, etc.

Compared to low-density linkage map, Hori *et al*.^49^ indicated that the higher-density linkage maps were more beneficial for QTL mapping, that is, the markers tightly linked to target QTL are more effective in marker assisted breeding^49^. Furthermore, it is possible to separate two closely linked QTLs by a high-density map with high resolution^49^. In this study, a high -density linkage map developed from an earlier study result^34^ was applied in QTL mapping. However, the confidence intervals of identified QTLs always were not narrow enough to predict candidate genes directly. Gene density is within a wide range of 0.5–10.7 genes per 100 kb in maize genome^50^. It is of great significance to study how to decrease the number of candidate genes located on confirmed QTL intervals. For the purpose, a common approach is to develop advanced generation population such as NIL(near isogenic line) population, thus to narrow down the QTL confidence interval significantly, even to clone the QTL or gene based on primary mapping results^51,52^. However, the construction of NILs is time - consuming and tedious, which limit the usage of large amount of objective QTLs in marker assisted breeding to a certain extent. In this study, the DEGs between parents were obtained from transcriptomic profiling of grains at different developmental stages and overlaid onto the confirmed QTL intervals to predict candidate genes for grain weight. Based on these DEGs, the candidate genes in the physical intervals of the ten confirmed QTLs (*qKW-7, qEW-9, qEW-10,qGWP-6, qGWP-8, qGWP-10,qGWP-11, qGWP-12, qGWP-13 and qGWP-14*) were decreased from over 1000 to 213 (Supplemental Table 3). Comparative genomics analysis was carried out to further predict candidate genes. Finally, a total of eight genes that might be involved in ubiquitin-proteasome degradation^30^, phytohormone signaling and transcription factor pathways were chosen as candidate genes controlling grain size and grain weight in maize.

In this study, a total of ten QTLs associated with grain weight were confirmed as stable QTLs, and eight candidate genes were predicted. All these results would be basis for cloning related functional genes and marker-assisted breeding.

## Methods

### Development of the F_2:3_ mapping population and field trails’ investigation

The F_1_ hybrid seeds were obtained from an intraspecific cross between two inbred lines SG5 and SG7 in Liupanshui, Guizhou in 2013 summer. There are significant differences in yield-related traits for the two inbred lines (Table 1). The obtained F_1_ seeds were also planted in Liupanshui, Guizhou in 2014 summer. A total of 199 F_2_ plants grew up from F_1_ seeds In November 2014, field trials were conducted at the Panxian Maize Breeding Station in Sanya, Hainan, China. The segregating population of 199 F_2:3_ lines, P_1_, P_2_ and F_1_ were all planted in field with a randomized block design of three replications, single-row plot with 15 plants, row spacing being 50 cm, and plant spacing being 35 cm. Seven agronomic traits including 100-kernel weight (KW), ear length (EL), ear diameter (ED), cob diameter (CD), kernel row number (KRN), ear weight (EW), and grain weight per plant (GWP) were investigated for the F_2:3_ population. Eight plants located in the middle of each plot were sampled for investigation after harvest and natural drying.

### High density linkage map, QTL analysis and validation

Methods of extracting genomic DNA, sequencing genotype, grouping sequence data, identifying single nucleotide polymorphisms (SNPs) and constructing high-density bin map were exhibited in our previous study^34^. QTL were detected by two methods: one is CIM method included in QTL Cartographer v. 2.5, and the other is genome-wide composite interval mapping (GCIM) method^53^. GCIM method was applied by using random model with maximum likelihood (ML) function. For CIM and GCIM mapping procedures, the walking speed was 1 cM and the logarithm of odds (LOD) threshold value was set up as 3.0. The position of a significant QTL was determined by its LOD peaks. The positive or negative additive effect of a QTL indicated that the increase or decrease in phenotypic value for a trait is provided by the alleles from SG5 or SG7. The graphic of QTLs positions on 10 maize chromosomes were drawn by MapChart 2.32 software^54^. The physical intervals between the QTLs identified in F_2:3_ population were compared with the intervals of QTLs mapped using a F_2_ population previously^34^, and Those QTLs both detected from F_2_ and from F_2:3_ populations show overlapped physical intervals will be regarded as confirmed QTLs.

### RNA sample preparation

The two maize inbred lines SG5 and SG7 were grown at the Panxian Maize Breeding Station, in Hainan, China in November 2016. Ear shoot were covered before silking. Some parental plants were hand pollinated when the length of corn silk was about 5 cm. Kernels were sampled from SG5 and SG7 on the 5-, 10, and 15-the days after pollination. Each sample consisted of three biological replicates in parallel. All samples were collected and frozen immediately with liquid nitrogen and stored in refrigerator at −80 °C. The total RNA of kernels at different growing stages was extracted by using TRIzol reagent (Invitrogen). One percent of agarose gels were used for RNA degradation and contamination. The NanoPhotometer® spectrophotometer (IMPLEN, CA, USA) was used for checking RNA purity. Qubit® RNA Assay Kit in Qubit® 2.0 Flurometer (Life Technologies, CA, USA) was used for measuring RNA concentration. The RNA Nano 6000 Assay Kit of the Bioanalyzer 2100 system (Agilent Technologies, CA, USA) was used for assessing RNA integrity. The Illumina NovaSeq platform was then applied to RNA-seq.

### Illumina sequencing and data analysis

A total of 18 samples with three repeats were collected and sequenced at the Illumina NovaSeq platform. Raw reads with fastq format were firstly handled by in-house perl scripts. Clean reads were then obtained after deleting reads containing adapter and ploy-N and removing reads of low quality in raw data. The GC content, Q20 and Q30 of the clean reads were calculated. High quality clean data were then carried out for further downstream analyzing. Maize reference genome and correlated files of gene annotation were downloaded directly from website (https://www.maizegdb.org/). Bowtie v. 2.2.3 and TopHat v2.0.12^39^ were used for building reference genome index and aligning paired-end clean reads to the reference genome, respectively. The reads mapped to every gene were counted by HTSeq v. 0.6.1.

For each gene, the expected number of Fragments Per Kilobase of transcript sequence per Millions base pairs (FPKM) was calculated by analyzing the gene length and mapped reads. FPKM is a widely accepted methodfor evaluating gene expression levels based on sequencing depth effect and gene length of the read count simultaneously^55^. The DEGSeq R package (v. 1.20.0) was used for analyzing differential expression genes between two conditions. The P value was corrected by using the Benjamini & Hochberg method. The threshold of corrected P-value 0.05 and log_2_ (Fold change) of 0.5 (absolute value) were considered as significantly differential expression. The GOseq R package was used for analyzing Gene Ontology (GO) enrichment of DEGs. The GO term with corrected P-value less than 0.05 was considered as DEG.

### Screening of candidate DEGs associated with QTLs for grain weight

In this study, the obtained RNA-seq data were used to explore DEGs between parental lines SG5 and SG7. Pair-wise comparison of transcriptomes between SG5 and SG7 was conducted for detecting DEGs. The DEGs were overlaid onto the physical intervals of confirmed QTLs to predict candidate genes for grain weight in maize.

## Supplementary information

Supplemental Tables and Supplemental Figures

Dataset 1
