# Supplementary material for: Mapping quantitative trait loci for yield-related traits and predicting candidate genes for grain weight in maize
Source: Sci Rep. 2019 Nov 6;9:16112. doi: 10.1038/s41598-019-52222-5 (PMC6834572; doi:10.1038/s41598-019-52222-5)
Supplement: Supplementary file 1 — Supplemental Tables and Supplemental Figures [file 41598_2019_52222_MOESM1_ESM.pdf]

Mapping quantitative trait loci for yield-related traits and predicting candidate genes for grain weight in maize

Yanming Zhao[1,] Chengfu Su[1*]

Supplemental Table 1. List of quality of output data for transcriptome analysis

Sample name	Raw reads	Clean reads	Error rate(%)	Q20(%)	Q30(%)	GC content(%)
P11_1	57557260	54965216	0.02	97.03	92.21	54.65
P11_2	52374646	50259848	0.02	97.06	92.31	54.57
P11_3	44355318	42655064	0.02	97.4	93.04	54.28
P12_1	59224744	57245328	0.02	97	92.17	53.94
P12_2	60433096	58163128	0.02	97.08	92.33	54.53
P12_3	60341270	58197780	0.02	97.19	92.6	54.05
P13_1	53550130	51998030	0.02	97.33	92.75	54.04
P13_2	52687798	50529740	0.02	97.35	92.93	54.68
P13_3	58001550	56009654	0.02	97.48	93.16	54.04
P21_1	42408646	40277620	0.02	96.82	91.79	54.14
P21_2	65111160	62763408	0.02	96.97	92.09	55.32
P21_3	55514856	53351498	0.02	97.16	92.53	55.42
P22_1	54426680	52448172	0.02	96.79	91.7	54.16
P22_2	46735930	44567324	0.02	97.11	92.42	54.27
P22_3	45001436	42392678	0.02	97.22	92.76	57.07
P23_1	51964988	50075872	0.02	97.4	93	54.41
P23_2	57230078	55362334	0.02	97.31	92.79	54.26
P23_3	53321240	51476196	0.02	97.36	92.89	54.69

P11_1, P11_2, P11_3 were samples obtained at 5 days after pollinating from SG5;
P12_1, P12_2, P12_3 were samples obtained at 10 days after pollinating from SG5;
P13_1, P13_2, P13_3 were samples obtained at 15 days after pollinating from SG5;
P21_1, P21_2, P21_3 were samples obtained at 5 days after pollinating from SG7;
P22_1, P22_2, P22_3 were samples obtained at 10 days after pollinating from SG7;
P23_1, P23_2, P23_3 were samples obtained at 15 days after pollinating from SG7;

Supplemental Table 2. Pearson correlation of RNA-seq data between samples

R^2	P11_1	P11_2	P11_3	P12_1	P12_2	P12_3	P13_1	P13_2	P13_3	P21_1	P21_2	P21_3	P22_1	P22_2	P22_3	P23_1	P23_2	P23_3
P11_1	1	0.985	0.969	0.862	0.894	0.913	0.715	0.8	0.755	0.876	0.882	0.883	0.804	0.798	0.818	0.711	0.667	0.665
P11_2	0.985	1	0.981	0.857	0.881	0.91	0.713	0.796	0.752	0.873	0.874	0.876	0.801	0.795	0.804	0.709	0.669	0.666
P11_3	0.969	0.981	1	0.82	0.845	0.904	0.688	0.769	0.727	0.853	0.858	0.858	0.77	0.761	0.772	0.678	0.645	0.641
P12_1	0.862	0.857	0.82	1	0.979	0.887	0.864	0.907	0.897	0.784	0.778	0.784	0.876	0.866	0.847	0.84	0.782	0.781
P12_2	0.894	0.881	0.845	0.979	1	0.907	0.831	0.9	0.872	0.803	0.805	0.807	0.855	0.847	0.87	0.814	0.74	0.742
P12_3	0.913	0.91	0.904	0.887	0.907	1	0.75	0.845	0.794	0.805	0.813	0.811	0.817	0.8	0.823	0.73	0.693	0.688
P13_1	0.715	0.713	0.688	0.864	0.831	0.75	1	0.944	0.982	0.661	0.654	0.66	0.778	0.76	0.722	0.88	0.867	0.878
P13_2	0.8	0.796	0.769	0.907	0.9	0.845	0.944	1	0.965	0.724	0.726	0.73	0.809	0.79	0.797	0.875	0.829	0.835
P13_3	0.755	0.752	0.727	0.897	0.872	0.794	0.982	0.965	1	0.693	0.687	0.692	0.799	0.781	0.759	0.884	0.855	0.863
P21_1	0.876	0.873	0.853	0.784	0.803	0.805	0.661	0.724	0.693	1	0.981	0.985	0.885	0.89	0.902	0.781	0.734	0.73
P21_2	0.882	0.874	0.858	0.778	0.805	0.813	0.654	0.726	0.687	0.981	1	0.993	0.872	0.871	0.904	0.774	0.724	0.722
P21_3	0.883	0.876	0.858	0.784	0.807	0.811	0.66	0.73	0.692	0.985	0.993	1	0.878	0.879	0.907	0.78	0.73	0.728
P22_1	0.804	0.801	0.77	0.876	0.855	0.817	0.778	0.809	0.799	0.885	0.872	0.878	1	0.984	0.923	0.899	0.875	0.867
P22_2	0.798	0.795	0.761	0.866	0.847	0.8	0.76	0.79	0.781	0.89	0.871	0.879	0.984	1	0.926	0.886	0.857	0.85
P22_3	0.818	0.804	0.772	0.847	0.87	0.823	0.722	0.797	0.759	0.902	0.904	0.907	0.923	0.926	1	0.852	0.769	0.77
P23_1	0.711	0.709	0.678	0.84	0.814	0.73	0.88	0.875	0.884	0.781	0.774	0.78	0.899	0.886	0.852	1	0.957	0.961
P23_2	0.667	0.669	0.645	0.782	0.74	0.693	0.867	0.829	0.855	0.734	0.724	0.73	0.875	0.857	0.769	0.957	1	0.989
P23_3	0.665	0.666	0.641	0.781	0.742	0.688	0.878	0.835	0.863	0.73	0.722	0.728	0.867	0.85	0.77	0.961	0.989	1

P11_1, P11_2, P11_3 were samples obtained at 5 days after pollinating from SG5;
P12_1, P12_2, P12_3 were samples obtained at 10 days after pollinating from SG5;
P13_1, P13_2, P13_3 were samples obtained at 15 days after pollinating from SG5;
P21_1, P21_2, P21_3 were samples obtained at 5 days after pollinating from SG7;

P22_1, P22_2, P22_3 were samples obtained at 10 days after pollinating from SG7;
P23_1, P23_2, P23_3 were samples obtained at 15 days after pollinating from SG7;

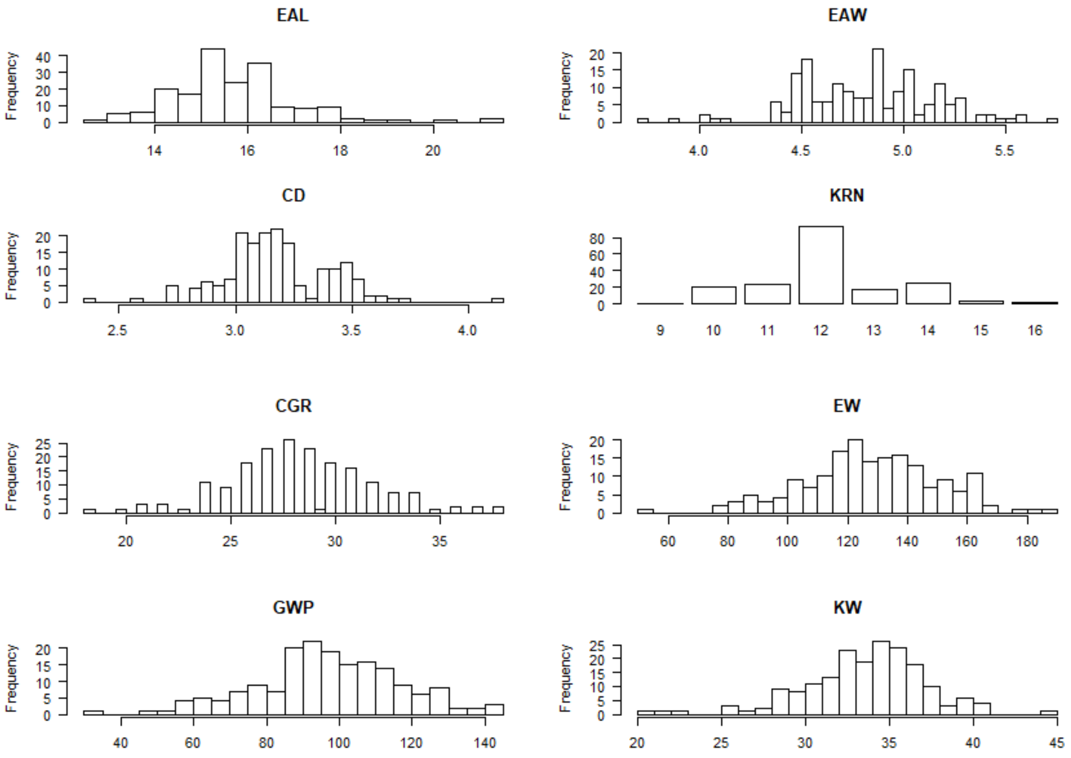

Supplemental Fig.1 Distribution of the ear length (EAL), ear width (EAW), cob diameter (CD), kernel row number (KRN), corn grains per row (CGR), ear weight (EW), grain weight per plant (GWP) and 100-kernel weight (KW) in the $F_{2:3}$ population derived from the cross of SG5 and SG7.

Supplemental Fig.2 Pearson correlation between samples

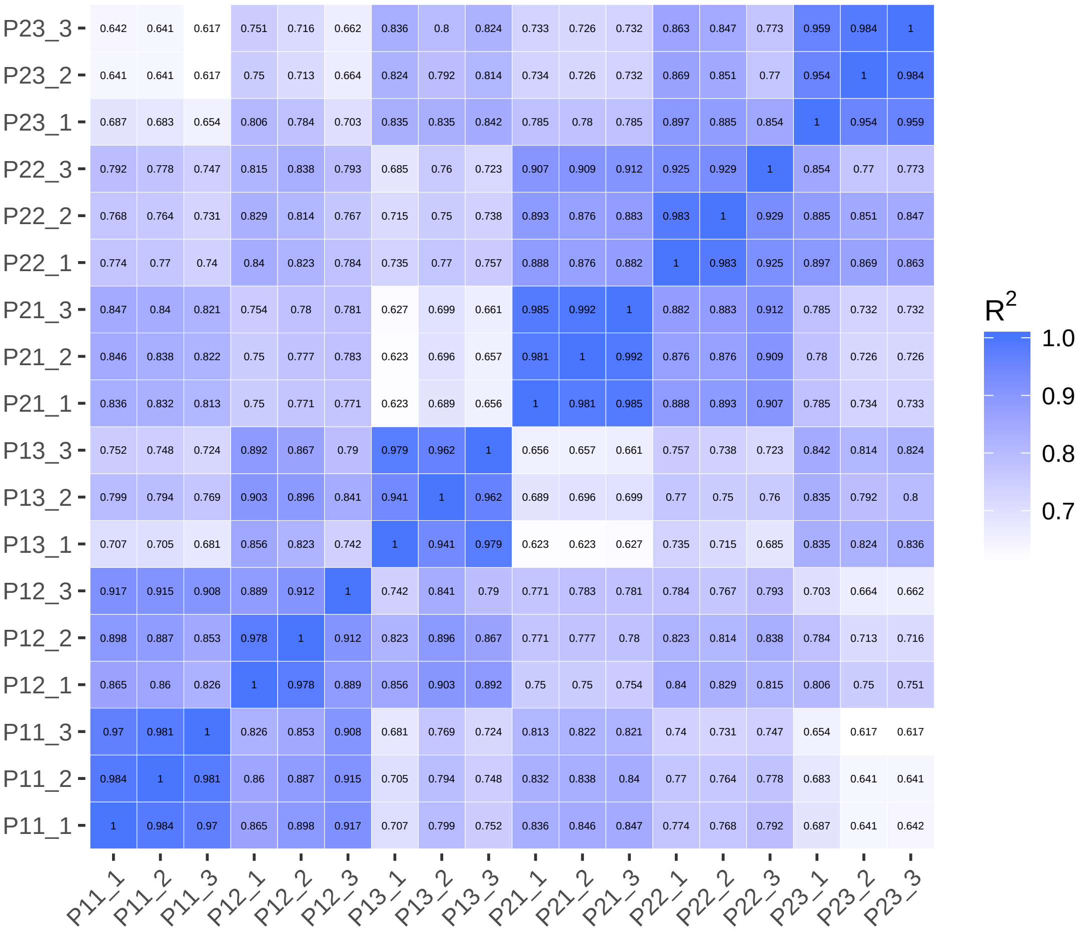

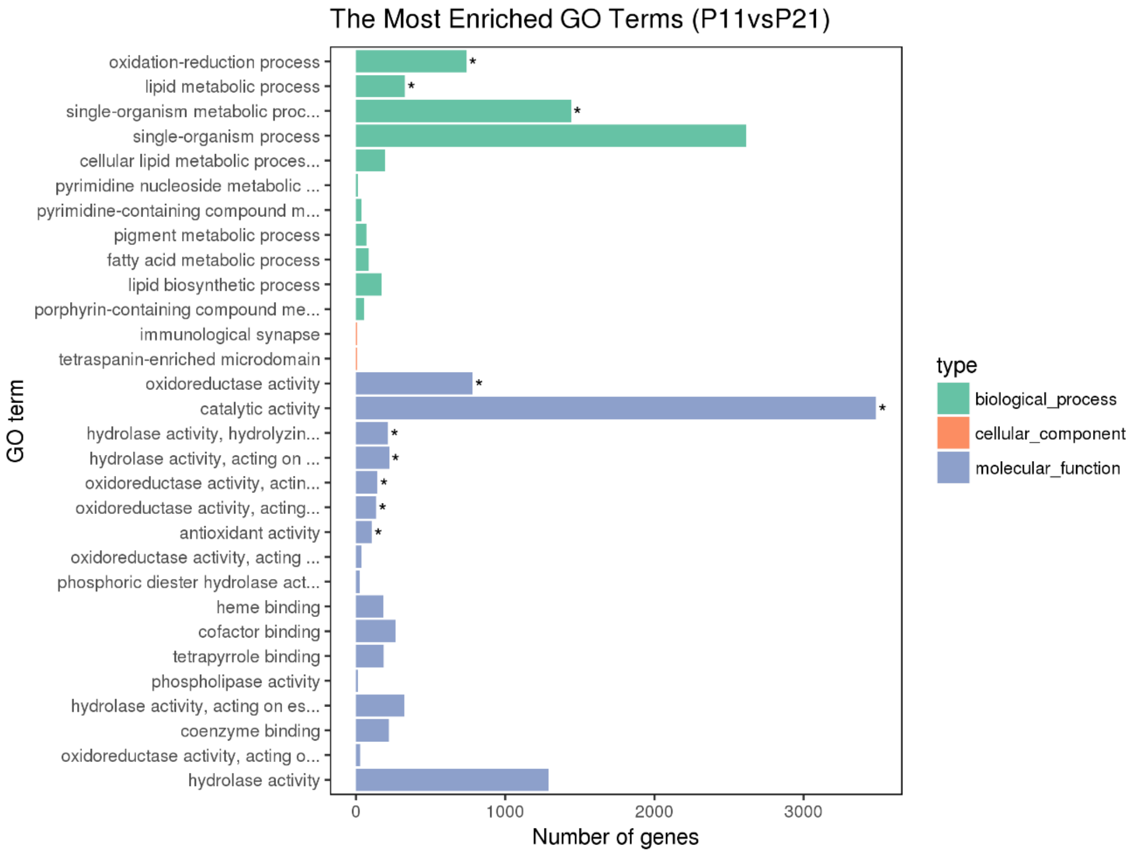

a: difference expression gene GO enrichment graph between P11 and P21

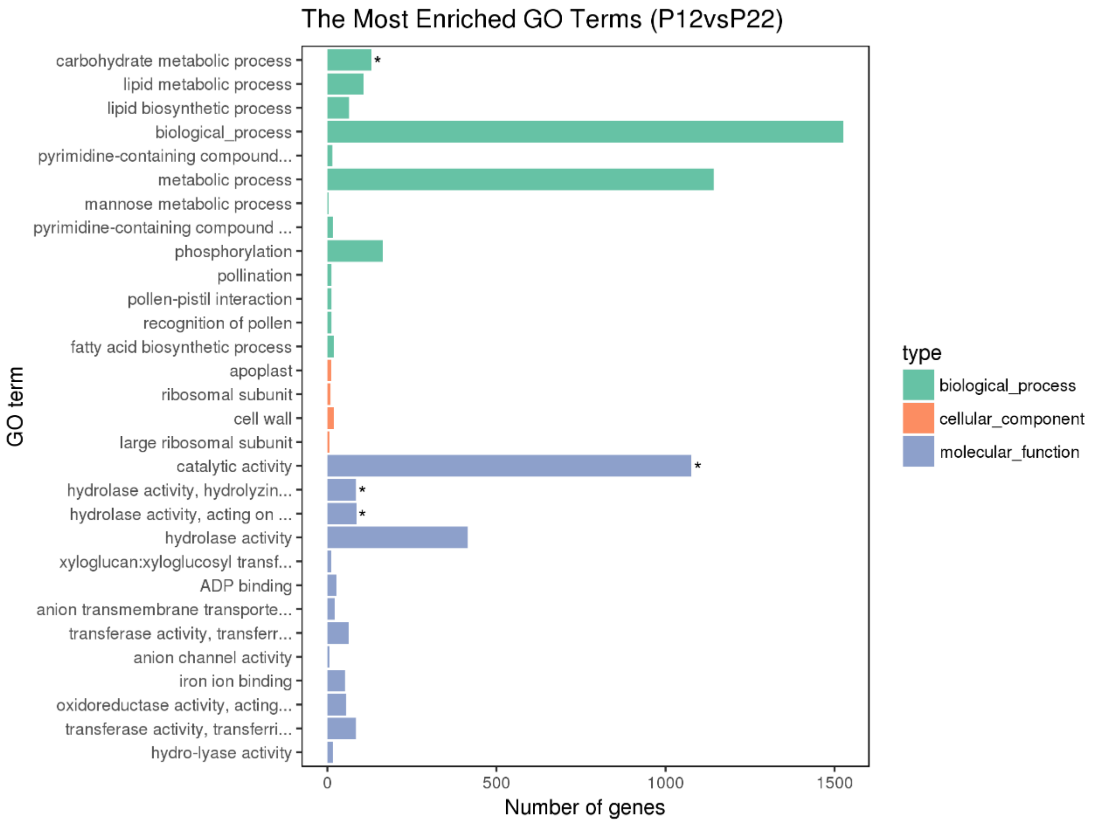

b: difference expression gene GO enrichment graph between P12 and P22

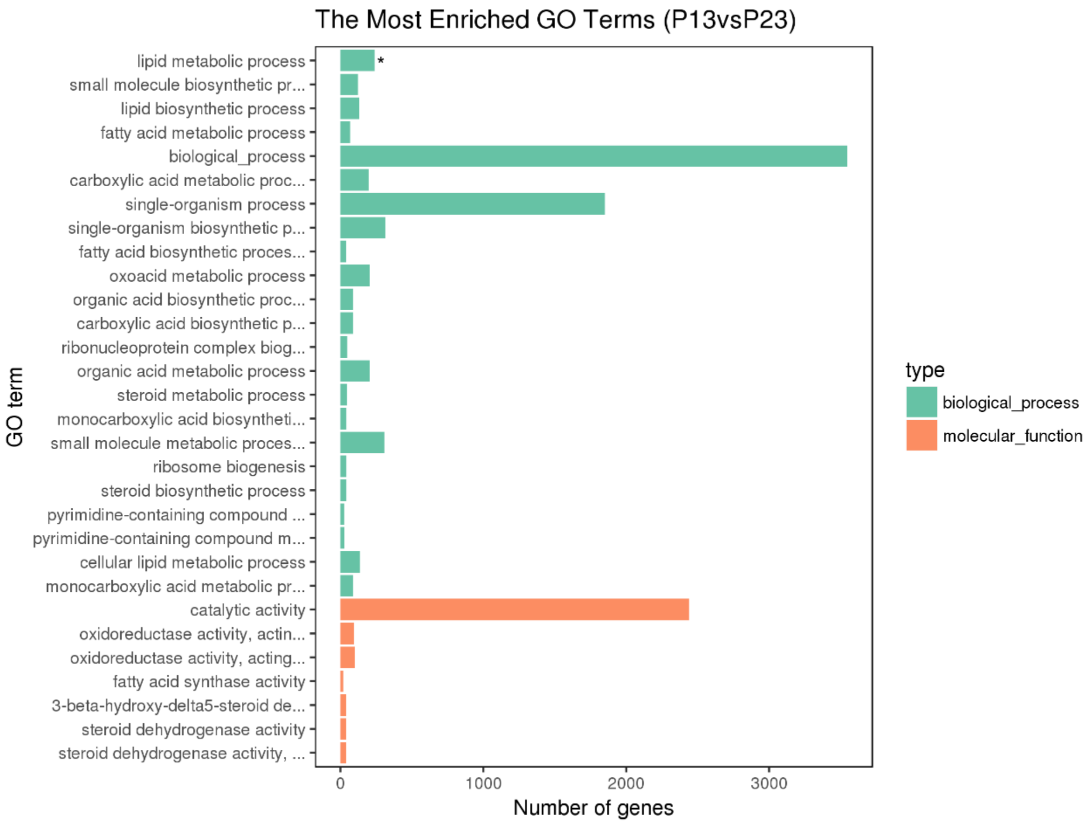

c: difference expression gene GO enrichment graph between P13 and P23

Supplemental Fig.3 Difference expression gene GO enrichment graph between parents SG5 and SG7 in different developmental stages. P11, P12, P13 indicated grains were sampled from maize inbred line SG5 while P21, P22, P23 indicated grains were sampled from maize inbred line SG7 at 5 days, 10 days, and 15 days, respectively after hand pollinating.
